# Uveitic band keratopathy: child and adult

**DOI:** 10.1186/s12348-015-0062-z

**Published:** 2015-11-21

**Authors:** Heloisa Nascimento, Mariana Kaori Yasuta, Maria Carolina Marquezan, Gustavo Henrique Araujo Salomão, Délia González, Claudia Francesconi, Cristina Muccioli, Rubens Belfort

**Affiliations:** Department of Ophthalmology and Visual Sciences, Federal University of Sao Paulo, Paulista School of Medicine, Sao Paulo, Brazil

## Abstract

**Background:**

Calcified band keratopathy is a chronic degenerative disease characterized by the deposition of gray to white opacity in superficial layers of the cornea that typically develops over months or years. It is associated with a variety of conditions, including chronic uveitis.

**Purpose:**

The objective of this study is to assess visual acuity and corneal changes in patients with band keratopathy secondary to uveitis who underwent phototherapeutic keratectomy (PTK).

**Setting:**

The place where this study was performed was in the Department of Ophthalmology and Visual Sciences, Federal University of Sao Paulo.

**Design:**

This is a retrospective study.

**Methods:**

Patients with uveitic band keratopathy were submitted to PTK. The PTK was performed using Allegreto Wave EX500, with the ablation area of 6 mm.

**Results:**

Twelve patients (13 eyes) diagnosed with band keratopathy secondary to chronic uveitis were analyzed. Of the 12 patients, 8 patients were female (66 %), aged 22 years (7–53 years). From the 12 patients (13 eyes) evaluated in this study, only one patient (one eye) did not have visual improvement, due to epithelial deposits 2 weeks after PTK, and all the others benefited with the procedure. In the children group, all eyes had visual improvement, and quantitatively speaking, the children had a more significant improvement than adults.

**Conclusions:**

PTK is a safe and effective procedure even for children. However, the improvement in visual acuity was restricted due to other ocular changes secondary to uveitis, such as cataract and retinal changes, or even the corneal irregularity.

## Background

Calcified band keratopathy is a chronic degenerative disease characterized by the deposition of gray to white opacity in superficial layers of the cornea, more frequently at interpalpebral zone that typically develops over months or years. Although it can occur as an idiopathic form, it is associated with a variety of conditions, including chronic uveitis [1, 2, 3, 4].

Treatment includes removal of calcium deposits with either ethylenediaminetetraacetic (EDTA), superficial keratectomy or phototherapeutic keratectomy (PTK), indicated for improvement of visual acuity and ocular discomfort caused by band keratopathy [5, 6].

The phototherapeutic keratectomy is an effective treatment for various disorders of the corneal surface, among them the band keratopathy. Irregularity of the corneal surface, epithelial instability, and superficial opacification may benefit from the procedure [1, 5]. This study was to assess visual acuity and corneal changes in patients with band keratopathy secondary to uveitis who underwent PTK.

## Methods

Retrospective study which analyzed patients with uveitic band keratopathy treated at the Uveitis Sector from the Department of Ophthalmology and Visual Sciences, Federal University of Sao Paulo, Paulista School of Medicine (São Paulo Hospital), between January and December 2013.

Patients underwent eye examination consisting of measuring visual acuity with pinhole, intraocular pressure (Goldmann tonometer), ectoscopy, biomicroscopy, and fundoscopy under mydriasis by indirect ophthalmoscopy lens of 20 diopters before and after 30 days of treatment. Those patients with a significant keratopathy in the visual axis, contributing to reduced vision, with pachymetry greater than 450 μm, with a maximum lesion depth of 100 μm, were chosen to undergo the PTK.

The PTK was the first option of treatment for these patients, all surgeries were performed under topical anesthesia, using Allegreto Wave EX500, with the ablation area of 6 mm. The procedure was performed aseptically. The corneal ablation is performed either by asking the patient to look at the light emitted by the device or manually focused laser. After using 70 to 80 % of the target ablation, the patient was examined at the slit lamp, to protect excess ablation of thin areas and decide on the need for additional treatment. At the end of the procedure, we put a bandage soft contact lens for 7 days and eye drops of antibiotic and corticosteroid [3, 5].

The protocol was approved by the Universidade Federal de São Paulo Research Ethics Committee and an informed consent was obtained.

## Results

Twelve patients (13 eyes) diagnosed with band keratopathy secondary to chronic uveitis were analyzed. Of the 12 patients, 8 patients were female (69.2 %), aged 22 years (61.5 % less than 18 years old, 23.1 % between 18 and 40 years old, and 15.4 % more than 40 years old).

One patient had posterior uveitis secondary to acute retinal necrosis (7.7 %), four panuveitis secondary to Vogt Koyanagi Harada syndrome (30.8 %), one intermediate uveitis (7.7 %), seven had anterior uveitis, three of them secondary to juvenile idiopathic arthritis (23.1 %), and four idiopathic anterior uveitis (30.8 %).

**Fig. 1:**
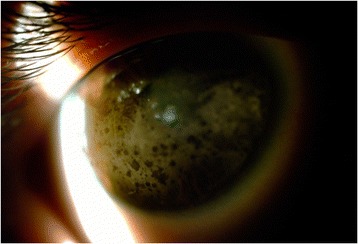
Before PTK

**Fig. 2:**
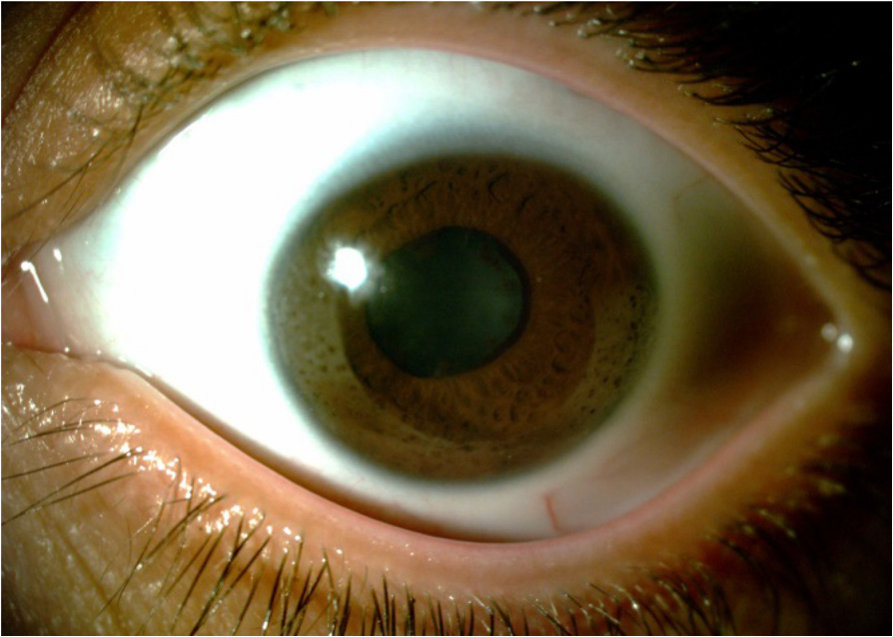
After PTK

**Fig. 3:**
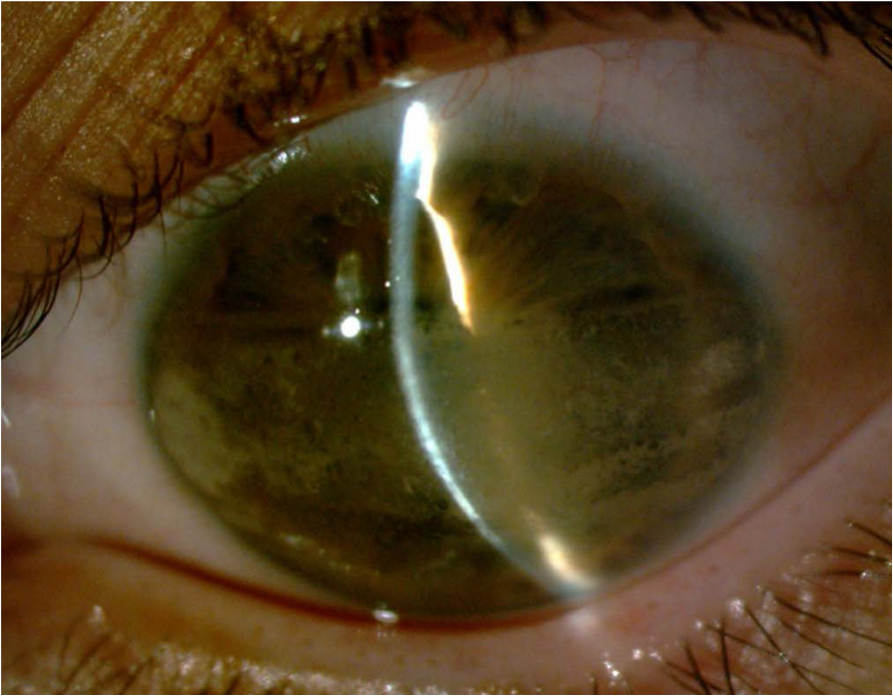
Before PTK

**Fig. 4:**
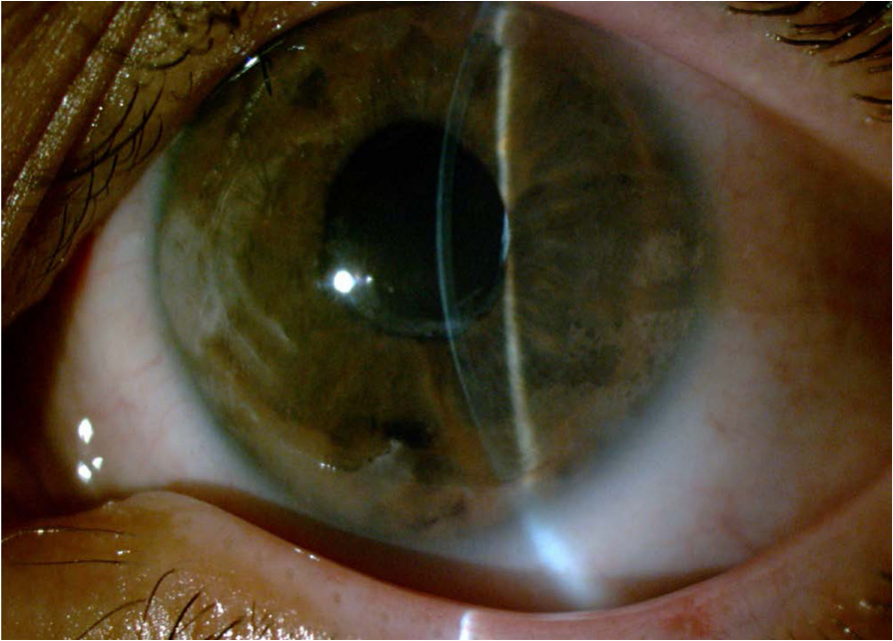
After PTK

Before PTK was performed, 38.5 % of the patients had visual acuity between 20/50 and 20/200, and the other 61.5 % had worse than 20/200. After the PTK, 23 % had visual acuity 20/40 or better, 31 % between 20/50 and 20/200, and 46 % had worse than 20/200 (Figs. 1, 2, 3, and 4).

**Fig. 5:**
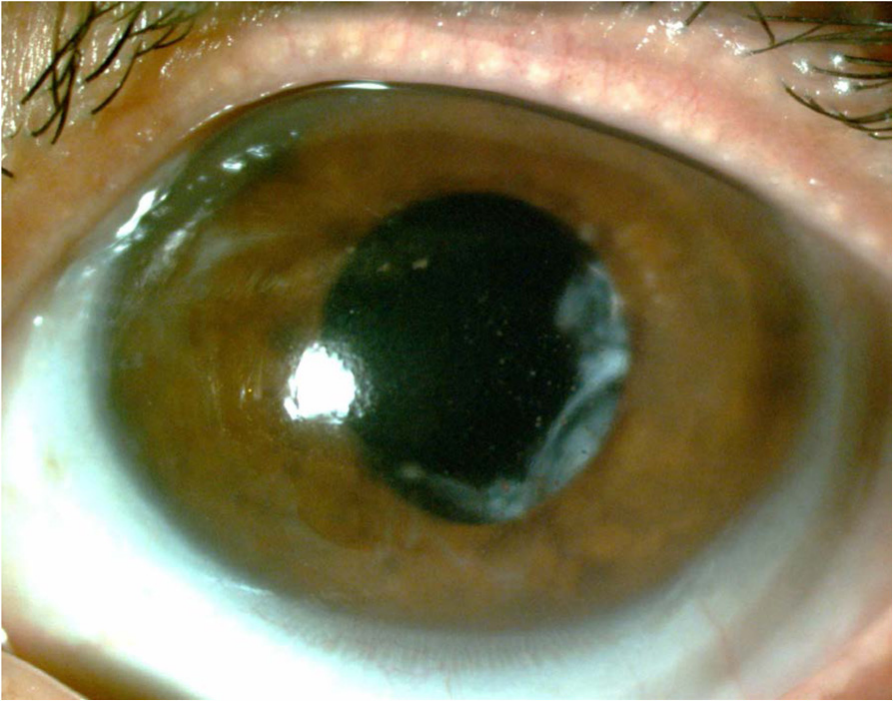
One day After PTK

**Fig. 6:**
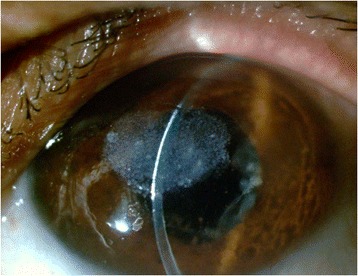
Two weeks after PTK

In the children group, all eyes had visual improvement. From the 12 patients (13 eyes) evaluated in this study, only 1 patient (one eye) did not have visual improvement, due to epithelial deposits 2 weeks after PTK (Figs. 5 and 6); all the others benefited with the procedure.

All the patients had central cornea involved and obtained central corneal clarity post treatment. Other relevant diseases detected were cataract and retinal changes.

Although the results were positive, there was no statistical significance between the variable final visual acuity and gender, age, or diagnosis (Tables 1 and 2).

**Table 1:** Children—gender, age, clinical diagnosis, and visual acuity before and after PTK

	Gender	Age (years)	Clinical diagnosis	VA before	VA after
1^a^	Female	12	Idiopathic anterior uveitis	20/100	20/32
2^a^	Female	12	Idiopathic anterior uveitis	20/160	20/25
3	Female	11	Anterior uveitis—JIA	20/80	20/50
4	Male	11	Idiopathic granulomatous anterior uveitis	Count fingers	20/400
5	Male	18	Idiopathic anterior uveitis	Light perception	Hand motion
6	Female	7	Vogt Koyanagi Harada Syndrome	Count fingers	20/200
7	Female	11	Vogt Koyanagi Harada Syndrome	Light perception	Count fingers
8	Female	7	Vogt Koyanagi Harada Syndrome	Hand motion	Count fingers

*VA* visual acuity, *JIA* juvenile idiopathic arthritis

^a^Same patient

**Table 2:** Adults—gender, age, clinical diagnosis, and visual acuity before and after PTK

	Gender	Age (years)	Clinical diagnosis	VA before	VA after
1	Male	28	Intermediate uveitis	20/60	20/30
2	Female	50	Acute retinal necrosis	20/100	20/160
3	Female	29	Anterior uveitis—JIA	Hand motion	20/400
4	Male	29	Anterior uveitis—JIA	Hand motion	Count fingers
5	Female	53	Vogt Koyanagi Harada Syndrome	20/400	20/200

*VA* visual acuity, *JIA* juvenile idiopathic arthritis

## Discussion

The mechanism of calcium deposition in the cornea is unknown, but it may result from precipitation left as tears evaporate, degeneration and necrosis from inflammatory diseases, changes in the pH, and the breakdown of phosphates [1, 2, 7]. This calcified material deposits at the Bowman’s membrane level [3, 4]. This study showed the benefits of excimer laser to achieve this layer, without manual error, in patients with chronic inflammatory diseases.

The excimer ablation allows precise tissue removal over wide areas of cornea without trauma to adjacent tissue, and this smoothing of the ocular surface after PTK may increase tear film stability, which may reduce the chances of recurrence and this regular tear film and cornea interface results in a better optical surface [2–4]. Stewart and colleagues showed that significant recurrence of band keratopathy was not seen in 2 years following PTK [5].

The main downside of the excimer laser ablation is that it does not discriminate between abnormalities, such as calcium, and normal stroma, and if the calcium deposits are not uniform, it can keep the irregular surface [2]. So, it is important to protect the healthy cornea of the laser and fill the irregularities with masking substances, such as balanced salt solution (BSS), avoiding this situation.

The main goal of PTK is to increase corneal transparency, providing visual rehabilitation, and prevent development of amblyopia in children [4].

The refractive changes after PTK can be variable, with myopic or hyperopic shifts, which can be corrected with spectacles or contact lenses [2, 6]. Other limit is the high cost of treatment [2].

## Conclusions

In summary, the PTK is a safe and effective procedure even for children. However, the improvement in visual acuity was restricted due to other ocular changes secondary to uveitis, such as cataract and retinal changes, or even the corneal irregularity. And the challenge is to keep inflammation under control because this chronic stimulus is an important risk factor for recurrence of band keratopathy.
